# State-dependent memory mechanisms insights from neural circuits and clinical implications

**DOI:** 10.3389/fncel.2025.1629796

**Published:** 2025-10-30

**Authors:** Yang Liu, Guohui Zhang, Rui Qi, Jie Ma, Jianguang Xu

**Affiliations:** 1 Department of Rehabilitation Medicine, Yueyang Hospital of Integrated Traditional Chinese and Western Medicine, Shanghai University of Traditional Chinese Medicine, Shanghai, China; 2 School of Rehabilitation Science, Shanghai University of Traditional Chinese Medicine, Shanghai, China; 3 Engineering Research Center of Traditional Chinese Medicine Intelligent Rehabilitation, Ministry of Education, Shanghai, China

**Keywords:** state-dependent memory (SDM), memory retrieval, memory encoding, memory storage, neural circuits

## Abstract

State dependent memory (SDM) occurs when memory retrieval varies with the individual's psychological and physiological state at encoding and recall. Growing evidence shows that internal states shape memory performance across all phases of memory. Examples include affective and physiological conditions, medication effects, and disease states. This review examines how these states affect encoding, storage, and retrieval. We argue that internal states modulate activity in brain regions involved in memory by altering neurotransmitter signaling and by inducing plastic organization of neural circuits and networks. We believe this perspective can guide personalized electrical neuromodulation and multimodal intervention strategies for memory disorders.

## 1 Introduction

State dependent memory (SDM) refers to better performance in memory retrieval when a person is in a similar state as when the memory was initially encoded (Bower, 1981). SDM often occurs under specific internal conditions, including anxiety, fear, depression, sleep, and states induced by alcohol or drugs (Ebrahimi-Ghiri et al., 2019). For example, when information is encoded in a state of depression, individuals more readily access memories congruent with that mood than unrelated memories (Ellwart et al., 2003; Everaert et al., 2022). Similarly, individuals with alcohol dependence may have difficulty recalling events learned while sober when intoxicated (Miller et al., 1978).

Memory is a dynamic cognitive process supported by neural circuits composed of excitatory and inhibitory neurons (Nelson and Valakh, 2015; Vogels et al., 2011). The balance between excitation and inhibition (E/I balance) within these circuits is tightly regulated by neurotransmitter systems, including glutamate, dopamine, noradrenaline, and others, and is critical for memory and cognitive function (Ghatak et al., 2021; Han et al., 2020; Takahashi, 2011; Parsaei et al., 2011). Emotional states can modulate this balance by influencing synaptic plasticity, thereby shaping memory processes (Han et al., 2020; Turkileri and Sakaki, 2017; Tanaka et al., 2000). For example, acute stress can disrupt the E/I balance, offering a circuit-level explanation for how internal states affect memory (Han et al., 2020).

Neural networks across brain regions govern memory and contribute to SDM. Key structures, including the hippocampal complex (Sumadevi, 2024), the amygdalar complex (McDonald, 2014), and the prefrontal cortex (PFC; Chafee and Heilbronner, 2022), play central roles in this process (Zarrindast and Khakpai, 2020). Memory emerges from widespread interactions among multiple regions that are coordinated through complex networks. In the fear-related network of mice, certain hub regions exhibit strong connectivity with other areas, and the strength of these connections can be dynamically modulated, affecting the functional state of the network (Vetere et al., 2017). The influence of internal states on memory depends on both circuit plasticity and mechanisms that reorganize networks, which in turn alter processing pathways and memory performance (Yang et al., 2006; Mohr et al., 2016; Hennings et al., 2019; Wen et al., 2021; Shine et al., 2016). Recent studies using a brain-wide neuron quantification toolkit have revealed different neuronal patterns across phases of aversive memory in males and females, although these differences were not reflected in behavior (Franceschini et al., 2023).

SDM may provide new insights for circuit-based therapeutic approaches, such as neurostimulation, in treating memory-related disorders. Neurodegenerative and psychiatric conditions, including Alzheimer's disease (AD), Parkinson's disease (PD), major depressive disorder (MDD), and anxiety disorders (ANX), frequently involve memory dysfunction (Demic and Cheng, 2014; Mioni et al., 2015; Lee and Fernandes, 2017; Vetere et al., 2025). Patients often exhibit memory deficits that are influenced by physiological or psychological states (Bronnick et al., 2007; Gisquet-Verrier et al., 2015; Radulovic et al., 2017; Huang and Li, 2021). For instance, individuals with PD commonly experience anxiety and depression, which can bias attention, impair working-memory updating and task-relevant encoding, and disrupt hippocampal-dependent retrieval, leading to difficulties in prospective memory such as remembering medication schedules (Yang et al., 2018; Hammarlund et al., 2024; Sumbul-Sekerci et al., 2022; Jia et al., 2018). These state-dependent effects have important implications for designing electrical neurostimulation protocols aimed at enhancing memory. Therefore, neurostimulation to enhance memory should be tailored to both the patients internal state and the targeted memory phase. While electrical neurostimulation is increasingly applied to memory disorders, evidence for its efficacy in improving memory or regulating internal states remains variable. Current interventions rely primarily on open-loop transcranial magnetic stimulation (TMS) and deep brain stimulation (DBS) targeting specific regions or networks (R. Rezai and Sharma, 2014; Marder et al., 2022). Neural responses to such stimulation show considerable variability across individuals (Wansbrough et al., 2024). Given that memory dysfunction in different disorders may be influenced by distinct internal states, there is a need for more precise, state-aware stimulation protocols.

In this review, we synthesize evidence for state dependence across encoding, storage, and retrieval and address the following: (i) state-dependent effects across these memory stages; (ii) supporting mechanisms within neural circuits and network plasticity; (iii) behavioral signatures and methods for assessing SDM; (iv) SDM manifestations in pathological conditions; and (v) circuit-based neuromodulation and intervention strategies for SDM. We argue that state-dependent mechanisms offer a promising framework for developing more precise and effective interventions to enhance memory and treat memory-related disorders.

## 2 State dependency in memory encoding, storage, and retrieval

This section reviews how internal physiological and emotional states influence memory across the phases of encoding, storage, and retrieval. Anxiety and acute stress can disrupt encoding by impairing attentional allocation and working-memory processes. Conditions such as sleep and stress modulate storage and consolidation, and they shape the durability and transformation of memory traces over time. Retrieval is particularly sensitive to the alignment between the emotional state at retrieval and that at encoding. Greater state congruence is typically associated with higher neural pattern similarity across phases and improved recall accuracy. To make phase boundaries explicit and to consolidate the physiological-process basis for our subsequent circuit-level discussion, we summarize state-dependent influences by memory phase in Table 1.

**Table 1:** State-dependent effects across memory phases: conditions, behavioral outcomes, and clarified onset-offset boundaries.

**Memory phase**	**Operational boundary (what marks the phase)**	**Condition/state**	**Effect on memory (behavioral)**	**Representative references**
Encoding	From stimulus onset → initial neural engram formation; attention allocation and working-memory updating	Trait worry/active worrying	Impairs working memory (WM) updating and attentional control; reduces encoding efficiency	Gustavson and Miyake (2015); Zhang et al. (2018)
		Anxiety/social anxiety	Prioritizes emotional/threat-related information; reduces neutral/task-relevant encoding	Yin et al. (2024); Shi et al. (2024a); Cooper and Langton (2006); Reeck et al. (2012); deBettencourt et al. (2021)
		Negative/threat-related stimulation	Narrows attentional scope under stress; weakens non-salient encoding	Cooper and Langton (2006); Reeck et al. (2012); Staal (2004)
		Acute stress (during encoding)	Enhances encoding of salient emotional material; weakens neutral encoding	Daffner et al. (2003); Clewett et al. (2018)
		Chronic stress/depression	Reduces WM updating; disrupts encoding efficiency	Zhang et al. (2018); Günseli et al. (2024)
		Post-traumatic stress state	Fragmented or incomplete sequence encoding	Kolk and Fisler (1995); Nadel and Jacobs (1998); Miyamoto et al. (2017); Chapman et al. (2023)
Storage/ consolidation	Consolidation (synaptic): post-encoding plasticity (hippocampus/cortex; long-term potentiation, LTP/long-term depression, LTD) before systems redistribution Consolidation (systems): gradual redistribution from hippocampus to neocortex; retrieval dependency shifts as systems consolidation proceeds	Sleep	Promotes slow-wave-driven synaptic plasticity; enhances consolidation vs. wake	Walker et al. (2002); Miyamoto et al. (2017)
		Alcohol intake	Disrupts LTP and synaptic transmission; impairs storage and later retrieval	Miller et al. (1978); White (2003)
		Acute stress (post-encoding)	Enhances emotional discrimination/“true memory” via cortisol and sympathetic activation; effects are short-lived/context-dependent	Smeets et al. (2008); Jiang et al. (2019); Cunningham et al. (2021); Payne et al. (2007); Corbett et al. (2017)
		Chronic stress/depression	Reduces hippocampal integrity; interferes with memory integration/consolidation	Zhang et al. (2024); Vetere et al. (2025)
		Trauma/PTSD	Fragmented consolidation; disorganized long-term storage	Kolk and Fisler (1995); Nadel and Jacobs (1998)
Retrieval	From cue onset → recollection/recognition decision; cue-driven reconstruction	Acute stress before recall	Reduces hippocampal and PFC activity; impairs recall accuracy	Oei et al. (2007); Larrosa et al. (2017)
		Cortisol elevation	Disrupts declarative and stimulus-response memory; produces transient forgetting	de Quervain et al. (1998); Guenzel et al. (2013); Tollenaar et al. (2008)
		Positive emotional states	Facilitate retrieval and bias recall toward positive memories; buffer stress	Xie and Zhang (2017); Speer and Delgado (2017)
		Mood congruence (encoding ↔ retrieval)	Matching emotional states increases recall accuracy and vividness	Ritchey et al. (2012); Christodoulou and Burke (2016)
		Pharmacological/ physiological state match	Drug/state congruence improves retrieval; mismatch reduces success (pharmacological SDM weakens over long delays)	Miller et al. (1978); Uner and Roediger (2022); Bardgett et al. (1996)

Each memory phase is defined by explicit onset-offset boundaries. Conditions are internal physiological or emotional states that modulate memory at that phase; “Effect on Memory” summarizes behavioral outcomes. References correspond to studies cited in the main text. For the major circuits/regions underlying these effects, see Supplementary Table 1.

### 2.1 State dependence in memory encoding

Memory encoding is the first stage of memory formation, during which information, such as sounds, images, or smells, is transformed into neural representations that can be processed and stored in long-term memory, often as sequential patterns of activity in the hippocampus (Levy and Wu, 1996; Lisman and Jensen, 2013; Kesner and Rolls, 2015). Memory encoding involves both the reception and the initial interpretation of information. The initial processing stage under negative emotional states is often fragile. For example, active worrying, a core component of anxiety, reduces working memory (WM) capacity, compromising the ability to handle multiple tasks or streams of information simultaneously (Sari et al., 2017).

Working memory is particularly critical during the early processing stage of encoding, as newly encoded information is retained for varying durations depending on processing depth. Sensory information is typically retained only briefly, with most dissipating during the sensory memory stage (Wang et al., 2016). Only a subset of information receives attention and is transferred to short-term or working memory, where it guides decision-making over minutes (Wang et al., 2016). During this stage, working memory plays an “updating” role, selecting and maintaining relevant information while removing outdated or irrelevant content (Ecker et al., 2010; Mastrangelo, 2024; Taylor et al., 2022).

Impaired working memory updating is observed in mood disorders and is associated with disruptions during the encoding phase, further reducing encoding efficiency (Zhang et al., 2018; Günseli et al., 2024). Research indicates that trait worry is linked to difficulties in updating working memory, even in non-threatening contexts (Gustavson and Miyake, 2015).

Anxious states prioritize threat-related information during early encoding, reallocating attention toward emotionally salient cues at the expense of other information, thereby reducing encoding efficiency (Yin et al., 2024; Shi et al., 2024a; deBettencourt et al., 2021). Selective attention shifts depending on task demands and emotional states, and studies of visual search demonstrate emotion-driven prioritization and stress-induced narrowing of attentional scope (Cooper and Langton, 2006; Reeck et al., 2012; Staal, 2004). This attentional bias can weaken the processing of task-relevant information, impairing memory encoding (Nie et al., 2024). Conversely, acute stress can enhance the encoding of salient material, highlighting state-dependent effects (Daffner et al., 2003; Clewett et al., 2018).

Fragmented sequence encoding has been observed in post-traumatic stress states (Kolk and Fisler, 1995; Nadel and Jacobs, 1998). Typically, theta (θ) oscillations coordinate neuronal activity to regulate memory formation, but extreme inhibitory states, such as during trauma, can weaken these oscillations, causing newly encoded information to enter subsequent stages in a fragmented form (Ji et al., 2024; Miyamoto et al., 2017; Chapman et al., 2023).

### 2.2 State dependence in memory storage

Following encoding, newly formed memories exist in a labile, temporary state, making them vulnerable to interference (Mosha and Robertson, 2016). Proactive interference occurs when previously learned information disrupts the learning of new, related material, while retroactive interference arises when new learning impairs retrieval of existing memories (Mercer and Fisher, 2022; Schubert, 2023). Consolidation transforms these fragile memory representations into stable long-term storage.

Physiological conditions, such as sleep, can significantly affect consolidation and memory storage (Xin and Yuan, 2015). For instance, Walker et al. (2002) found that sleep enhances learning compared with wakefulness over the same interval, a phenomenon known as sleep-dependent memory consolidation. During sleep, synchronized neuronal oscillations, including slow waves (0.5–4 Hz), regulate synaptic strength and facilitate memory consolidation (Miyamoto et al., 2017).

Alcohol can disrupt memory storage by affecting synaptic plasticity, causing neuronal hyperpolarization, impaired signal transmission, and reduced long-term synaptic connectivity, which together interfere with proper memory storage and retrieval (Miller et al., 1978; White, 2003).

Stress effects on memory storage are not uniformly negative. Acute stress can enhance discrimination of related memories and improve true memory performance during consolidation, effects that are closely linked to stress-induced cortisol and sympathetic activity (Smeets et al., 2008; Jiang et al., 2019; Cunningham et al., 2021). However, these enhancements are often short-lived or context-dependent, for example, favoring emotional over neutral memory during early consolidation (Payne et al., 2007; Corbett et al., 2017). Additionally, hippocampal dysfunction in depression and trauma-related memory fragmentation in PTSD can disrupt consolidation and memory organization, discussed further in Section 5.

### 2.3 State dependence in memory retrieval

Memory retrieval involves recalling stored information and accessing established memory traces (Savarimuthu and Ponniah, 2023). Successful retrieval depends on the fidelity of neuronal discharge sequences formed during encoding (Vaz et al., 2020), with greater similarity between encoding and retrieval activity facilitating recall (Ritchey et al., 2012). When emotional states during retrieval match those during encoding, information is more readily accessed, whereas state-inconsistent memories may be overlooked or forgotten (Christodoulou and Burke, 2016).

Acute stress prior to retrieval typically impairs memory, as demonstrated by functional magnetic resonance imaging (fMRI) studies showing reduced hippocampal and PFC activity when stress occurs after encoding but before retrieval (Oei et al., 2007; Larrosa et al., 2017). Stress can also alter synaptic plasticity by inducing long-term depression (LTD) and disrupting hippocampal circuit interactions, leading to transient or persistent forgetting (Diamond et al., 2005; Yang et al., 2006). Cortisol elevations further impact both hippocampus-dependent declarative and striatum-dependent stimulus-response memory, potentially compromising long-term memory (de Quervain et al., 1998; Tollenaar et al., 2008; Guenzel et al., 2013).

Emotional memory retrieval is particularly relevant for affective disorders, which are often characterized by biases toward negative memories (Paunovic et al., 2002; Buchanan, 2007; Cohen and Kahana, 2022). Individuals with depression or in irrelevant contexts may ruminate on negative memories, limiting their ability to respond to current challenges (Gotlib et al., 2004; Leyman et al., 2006; Alipour et al., 2025). Conversely, strategies that promote retrieval of positive emotional memories can buffer stress and modulate affective states (Speer and Delgado, 2017).

## 3 The role of circuits and networks in SDM

This section outlines how neural circuits and large-scale networks support SDM. At the circuit level, interactions among the hippocampus, PFC, amygdala, and other interconnected regions shape state-dependent encoding, storage, and retrieval. At the systems level, large-scale networks organize attention, control, and internal mentation, and shifts in arousal and affect can rebalance these networks and change memory performance. Supplementary Table 1 summarizes phase-specific neural substrates, highlighting representative circuits and networks implicated in SDM.

### 3.1 Dynamic regulation of brain areas and circuits in SDM

During memory encoding, the bidirectional functional connectivity between the hippocampus and PFC is a key neural circuit (Das and Menon, 2024; Su et al., 2024). The hippocampus is responsible for integrating information from different brain regions into a complete memory representation (Marr, 1971; Eichenbaum, 2004), whereas the PFC is involved in memory encoding (Fletcher, 1998) and can influence the computations of the hippocampus during memory encoding by providing top-down state signals (Nyberg et al., 2019). The hippocampus interacts with the PFC to integrate old and new knowledge, guiding the encoding of new memories (Preston and Eichenbaum, 2013; Sekeres et al., 2024). During encoding, the amygdala supports state-dependent processing of emotional information (LaBar and Cabeza, 2006). The basolateral amygdala (BLA; Patel et al., 2016) contributes to encoding of salient negative information across internal states, especially in the recognition and response to emotions such as fear and anxiety (LeDoux, 2003). During this process, the hippocampal complex contributes contextual representations and interpretive frameworks, thereby modulating the amygdala's response to emotionally salient stimuli (Phelps, 2004). However, the amygdala and hippocampus do not synergize in all types of memory. During the encoding of associative memory, an increase in amygdala activity, does not disrupt hippocampal association-memory processes, although it impairs emotional associative memory (Madan et al., 2017). This indicates that under negative emotions, the amygdala does not inhibit the associative encoding function of the hippocampus.

During memory storage and consolidation, the PFC supports retention by regulating posterior sensory representations rather than storing content itself (Lara and Wallis, 2015). It monitors and organizes memory and, together with the hippocampus, forms a schema network that integrates new information into existing knowledge (Simons and Spiers, 2003; Maviel et al., 2004; Frankland and Bontempi, 2005; Khan et al., 2024). The central amygdala (CeA) contributes to early consolidation of fear memory and is not merely a downstream relay of the basolateral complex (Pitts and Takahashi, 2011; Kim et al., 2017). In mice, fear learning and consolidation involve engagement of a distributed thalamic-hippocampal-cortical network, which shows small-world organization and supports long-term stability (Wheeler et al., 2013). In humans, fear-memory storage is associated with increased connectivity between the amygdala and dorsal anterior cingulate cortex (dACC) and between the hippocampus and insula, together with decreased connectivity between the amygdala and medial PFC (mPFC; Feng et al., 2013). Stressful states can compromise the transfer of information into long-term memory, and chronic stress can inhibit hippocampal long-term potentiation (LTP; Lynch, 2004; Schwabe et al., 2009; Zerbes and Schwabe, 2019).

Interactions among the amygdala, hippocampus, and mPFC support memory retrieval, especially for emotional memories (Buchanan, 2007; Daselaar et al., 2008; Oztekin et al., 2009; Murty et al., 2010). Human studies show that the amygdala participates in emotional arousal. Arousal increases retrieval-related activity in the amygdala and the medial temporal lobe (MTL; Squire and Zola-Morgan, 1991) during long-term memory (Dolcos et al., 2005; Madan et al., 2017). Retrieval engages multiple circuits, including interactions between the postsubiculum and the hippocampus and between the amygdala and the hippocampus via the lateral amygdala (Richter-Levin, 2004; Sotres-Bayon et al., 2012; Wang et al., 2023). In conditioned fear, inputs from the BLA and the ventral hippocampus (vHPC) oppositely modulate the prelimbic (PL) cortical circuitry and shape fear expression (Sierra-Mercado et al., 2011; Sotres-Bayon et al., 2012). The anterior cingulate cortex (ACC) participates in remote contextual fear retrieval, although its dynamic links to other circuits remain unclear (Frankland et al., 2004). Successful retrieval of extinction memory suppresses the original fear response, yet stress impairs extinction learning (Akirav and Maroun, 2007). During extinction, the infralimbic (IL) cortex to BLA pathway inhibits fear expression, and an IL to thalamic nucleus reuniens (Re) to BLA pathway is engaged (Silva et al., 2021; Vafaei et al., 2022; Li et al., 2025). Re links the mPFC and the hippocampus, supports spatial working memory and executive functions, and may be a target for DBS in AD (Viena et al., 2018; Shoob et al., 2023).

Multiple neurotransmitters that participate in signal transmission in memory-related brain regions can also modulate memory and states, including the predominantly excitatory glutamatergic system, the dopaminergic system (Kong et al., 2024), the noradrenergic system (Downs and McElligott, 2022), and the predominantly inhibitory GABAergic system (Russek, 2006). Earlier works showed that these neurotransmitter systems can alter the balance of excitation and inhibition (E/I balance) in neurons during information transmission (Ghatak et al., 2021), which can affect memory performance under certain conditions, such as acute stress (Han et al., 2020). The E/I balance is crucial for maintaining neuronal stability and normal brain function and disruptions in this balance are associated with various neuropsychological disorders (Yizhar et al., 2011; Duma et al., 2024; Racz et al., 2025).

Gamma-aminobutyric acid (GABA) is an important inhibitory neurotransmitter in the central nervous system (CNS; Russek, 2006) that plays a role in maintaining the order of information transmission in the E/I balance (Rideaux et al., 2022). GABA-A receptors in the dorsal hippocampus (Wang et al., 2015) are implicated in various neurodegeneration diseases (Rissman and Mobley, 2011) and participate in Lithium-induced SDM in mice (Parsaei et al., 2011). Lithium is a commonly used mood stabilizer for treating human bipolar disorder (Rybakowski, 2014). In addition, GABA receptors are also believed to play a role in the acquisition, consolidation, and extinction of fear memory (Dubrovina, 2017).

The glutamatergic system, through α-amino-3-hydroxy-5-methyl-4-isoxazole-propionic acid receptors, is involved in enhancing synaptic plasticity that underlies fear memory formation (Gonzalez and Jayaraman, 2024). This process may help explain how the hippocampus encodes and stores memories related to fear and avoidance. The N-methyl-D-aspartate (NMDA) receptor, another key component of the glutamatergic system, plays a crucial role in the reconsolidation of aversive memories within the amygdala complex (Garcia-delaTorre et al., 2014). Another important neurotransmitter related to aversive memory is histamine (Fabbri et al., 2016). Specifically, it has been shown that depletion of histamine impairs the retrieval of inhibitory avoidance memory in rats (Fabbri et al., 2016), especially when the depletion occurs in the hippocampus or BLA, which damages long-term memory (Benetti et al., 2015). However, when the hippocampus is damaged, restoration of histamine signaling in the BLA can improve memory recovery, suggesting that in damaged brain structures, the histaminergic system's regulation of aversive memory can be compensated through other pathways.

Emotional experiences may be better remembered, partly because adrenergic receptors also influence synaptic plasticity (Katsuki et al., 1997) and enhance memory consolidation (Roozendaal and Hermans, 2017). However, this process is complex, as emotions do not always enhance memory (Madan et al., 2017; Turkileri and Sakaki, 2017). Under acute stress, the noradrenergic system is widely activated, which enhances emotional memories (e.g., fear) and is implicated in anxiety-related arousal (Goddard et al., 2010; Tanaka et al., 2000). In other laboratory studies, chronic stress was found to significantly increase the norepinephrine (NE) transporter and tyrosine hydroxylase, both of which are involved in the synthesis and release of noradrenaline, impairing working memory (Miner et al., 2006; Lee and Goto, 2015). Repeated stress in rats can lead to impairments in spatial and emotional memory due to activation of α1 adrenergic receptors (Faraji et al., 2023).

Dopamine (DA) plays a crucial role in anxiety and fear (de la Mora et al., 2010; El Mansari et al., 2010; Bannon et al., 2020). DA regulates the formation of memories by affecting synaptic plasticity in the hippocampus (Jay, 2003), specifically by enhancing or inhibiting hippocampal LTP and LTD (Tsetsenis et al., 2023). There are five subtypes of dopamine receptors, D1–D5 (Bannon et al., 2020). Among them, the D1 family (D1/D5 receptors), when activated, plays a crucial role in long-term synaptic plasticity in various regions of the hippocampus (Hansen and Manahan-Vaughan, 2012) and may support the dopaminergic modulation of persistent memory (Nagai et al., 2007). Hagena and Manahan-Vaughan (2016) found that the use of D1/D5 receptor antagonists in the hippocampus of laboratory rats significantly impaired LTP and LTD induced by patterned incoming stimuli or new spatial learning. This finding suggests that D1/D5 receptors play an important role in either enhancing or suppressing synaptic plasticity (Lemon and Manahan-Vaughan, 2006). In specific circumstances, the activation of these receptors can trigger the release of DA in the hippocampus, which promotes experience-dependent memory encoding (Hagena and Manahan-Vaughan, 2016) and consolidation of different tasks (Furini et al., 2014). The D1/D5 receptors-induced release of DA in the PFC further promotes long-term retention of recognition memory (Nagai et al., 2007).

### 3.2 Plasticity of neural networks and SDM

Shi et al. (2024b) demonstrated that whole-brain integration during the memory encoding phase is positively correlated with memory performance, whereas during retrieval, cross-module recruitment of visual and somatomotor networks contributes to memory performance. These findings suggest that different memory stages rely on distinct patterns of network reorganization to meet task demands, highlighting the inherent plasticity of neural networks.

The brain is capable of rapidly reorganizing neural circuits through synaptic modifications occurring within hours, allowing swift adaptation to new experiences and the formation of novel functional subnetworks (Glasser et al., 2011). Such structural changes not only modify local circuits but also enhance global efficiency, particularly within prefrontal cortical networks, thereby reconstructing retrieval pathways for memories (Le Bé and Markram, 2006; Mohr et al., 2016).

Neural circuits flexibly reconfigure according to task demands across different contexts (Trambaiolli et al., 2024). Human fear-extinction studies reveal cross-regional connectivity that extends beyond “classical fear-related” areas, encompassing large-scale networks such as the Default Mode Network (DMN), Ventral Attention Network (VAN), and Frontoparietal Network (FPN), whose functional connectivity dynamically changes during extinction (Wen et al., 2021). Rodent studies show that recall of fears of varying intensity triggers marked changes in circuit coupling (Haubrich and Nader, 2023). Under mild fear, the amygdala (BLA/CeA) acts as a central hub, with the infralimbic cortex (IL) bridging the amygdalar module to the retrosplenial cortex (RSC) and hippocampus. Under severe fear, the network fragments, with the amygdala becoming disconnected from other regions, illustrating state-dependent plasticity. Recall networks are integrated under mild fear but fragment under severe fear, providing a network-level perspective that contextualizes disorders of fear dysregulation such as PTSD and social anxiety disorder.

Anxiety also induces plastic changes in coupling among large-scale systems. fMRI studies report altered connectivity between the DMN and the salience network (SN; Li et al., 2023; Willinger et al., 2024), as well as between the DMN and the dorsal attention network (DAN; De la Peña-Arteaga et al., 2024). DMN dysfunction can contribute to emotional disorders such as anxiety (Zhao et al., 2007). The SN identifies and highlights salient stimuli in the environment, and its overactivation can lead to excessive vigilance and heightened attention to threat (Han et al., 2023). The DAN demonstrates increased sensitivity to threatening stimuli in anxiety, influencing memory performance on attention-dependent tasks (Gao et al., 2023).

Anxious states may lead to excessive processing of threatening information and overemphasis on errors, increasing the likelihood that negative information enters working memory (Grant and White, 2016; Bar-Haim et al., 2007). These network-level shifts are associated with reduced perceptual efficiency and impaired control of worry, and can influence memory across multiple stages (Kim, 2010; Yuan et al., 2021; Xiong et al., 2020; Mallas et al., 2020). Studies on trait anxiety indicate that current emotional states modify the network expression of the trait (De la Peña-Arteaga et al., 2024). When state anxiety is regulated, trait-related associations are focal within the DAN, ventral DMN (vDMN), and auditory network (AN); without such regulation, additional clusters appear in other networks. These patterns reflect a reconfigurable network architecture rather than isolated regional effects, linking SDM to large-scale network plasticity.

## 4 Understand SDM at the behavioral level

Behavioral assessment of SDM involves three complementary approaches. Task paradigms quantify working and prospective memory under varying cognitive loads, emotion-memory paradigms examine how affect influences memory control and retention, and clinical scales capture state-related changes in cognitive function. Together, these methods provide a comprehensive picture of how internal states shape memory accuracy, accessibility, and overall availability.

### 4.1 Behavioral differences in SDM

Emotional states act as internal contexts that can strongly influence memory retrieval (Madan et al., 2017). Reinstating negative emotional contexts during retrieval often enhances memory accuracy, while reinstating positive contexts increases the likelihood of successful recall (Xie and Zhang, 2017). This demonstrates that emotional states affect both the accessibility and quality of retrieved memories. These findings align with recent research on emotional dynamics and temporal memory (Wang and Lapate, 2025), showing that shifts from neutral to negative emotions can produce a perceived expansion of time. This “time expansion effect” may serve an adaptive function by enhancing the encoding and retention of emotionally significant events.

Beyond emotional states, physiological factors such as fatigue also impact memory. Fatigue is associated with reduced accuracy and impaired retrieval (Fan et al., 2017), which can lead to information loss and retrieval bias. Such impairments may result from reduced attentional resources under fatigue or high stress, ultimately diminishing cognitive efficiency and memory quality (Stephenson et al., 2019; Kayvani et al., 2023).

### 4.2 Behavioral methods for assessing SDM

Several behavioral tasks have been developed to evaluate memory performance under different internal states. The n-back test, for example, measures working memory capacity and efficiency (Jaeggi et al., 2010) and can track performance changes under varying cognitive loads (Frost et al., 2021). Under high stress or anxiety, recall of neutral information often declines, while memory for emotional material may improve. Fatigue can make “remember” cues harder to execute and increase sensitivity to “forget” cues.

Prospective memory (PM) tasks provide another approach to assessing SDM. PM refers to the ability to remember and carry out intended actions in the future (Arnold et al., 2014; de Mendonça et al., 2018). By manipulating the relationship between PM goals and ongoing tasks, studies have shown interactions between cognitive load and attentional focus, with the longest response times occurring under conditions of both high load and strong focus (Cantarella et al., 2023). In clinical settings, PM failures, such as missed medication in PD, illustrate the impact of internal states on memory under varying cognitive demands.

Emotion-memory paradigms are central to SDM assessment because emotional states influence both encoding and retrieval. The directed forgetting paradigm, for instance, evaluates how state interference affects memory control by comparing forgetting for emotional vs. neutral materials (Wierzba et al., 2018). Emotional induction tasks, using visual images or music, test how affective cues modulate memory performance (Diener et al., 2023). Emotional working memory tasks, such as the two-back emotional working memory task, assess how affective content influences the maintenance and updating of information (Zhang et al., 2022; Yavas, 2024).

Clinical scales complement laboratory tasks by capturing broader cognitive and emotional effects on memory. The Hamilton Anxiety Scale (HAS) includes cognitive items that reflect attention, memory, tension, worry, fatigue, and somatic symptoms (Gjerris et al., 1983). Although the scale does not directly measure emotion-memory interactions, anxiety as a state can disrupt memory processing, leading to reduced performance on cognitive tests that depend on attention and executive function (Isakulyan and Marachev, 2024). Broader assessments, such as the Montreal Cognitive Assessment (MoCA) and the Mini-Mental State Examination (MMSE), measure attention, executive function, and memory. Combined with behavioral tasks, these scales provide multi-dimensional, indirect measures of how emotional states influence cognition. For example, patients with anxiety often score lower on the MoCA compared with non-anxious controls, suggesting that heightened anxiety can impair attention, executive function, and memory (Liu et al., 2016; Li et al., 2017).

## 5 SDM regulation in pathological conditions

Neurodegenerative and psychiatric disorders show memory impairments that depend on internal states. These impairments involve alterations in key brain regions, circuits, neurotransmitter systems, and network connectivity. This section discusses how internal states influence memory in each condition and how these insights can guide targeted interventions and treatment strategies.

### 5.1 State-dependent changes in neurodegenerative diseases

AD involves progressive decline in contextual, spatial, working, and semantic memory (Arlt, 2013). Patients often struggle to recall recent experiences, complete complex tasks, understand certain vocabulary and concepts, and navigate their environment (Arlt, 2013). Multiple brain regions, particularly the hippocampus and cortex, degenerate, impairing structures, function, and neural circuits (Vetere et al., 2025). Early hippocampal atrophy may heighten sensitivity to stress, accelerating memory decline (Bierman et al., 2009). Patients also often show difficulties in recognizing and regulating emotions, including impaired emotional discernment, anxiety, and depressive symptoms (Sinclair et al., 2023). These neuropsychiatric signs may signal early social behavior problems (Strijkert et al., 2023) and affect quality of life (Martinez et al., 2018). Stress can further disrupt emotional processing, related to anxiety and cortisol levels (Gómez-Gallego and Gómez-García, 2018). Sleep disturbances, including fragmented nighttime sleep, excessive napping, and disrupted sleep-wake cycles, are common (Peter-Derex et al., 2015) and can worsen cognitive and emotional deficits (Cui et al., 2025). Environmental factors, such as pollutants, lifestyle, and diet, also contribute to AD risk (Saragea, 2024). These state-dependent effects, where impaired connectivity impacts both new memory encoding and retrieval, are especially evident with hippocampal and cortical decline (Dufort-Gervais et al., 2019; Silva and Martínez, 2023).

PD is another prevalent neurodegenerative disorder (Jagadeesan et al., 2017). Its pathology is mainly linked to dopaminergic neuron loss in the substantia nigra pars compacta, resulting in dopamine depletion in basal ganglia circuits (Jagadeesan et al., 2017; Latif et al., 2021). Working memory deficits are common in PD, manifesting as difficulty maintaining and manipulating information (Muslimovic et al., 2005; Giehl et al., 2020), linked to prefrontal-striatal circuit dysfunction (Gruber et al., 2006; Fallon et al., 2017; Lee et al., 2025). Unlike AD, where integrating new information is the main issue, short-term memory problems in PD often present as increased random guessing, attentional fluctuations, and executive dysfunction (Zokaei et al., 2020). Memory consolidation and attentional filtering can also be impaired (Lee, 2023). Cognitive deficits extend to decision-making, executive control, and cognitive flexibility (Pagonabarraga and Kulisevsky, 2012; Smith et al., 2020). In PD with mild cognitive impairment, widespread deficits can impair prospective memory, such as remembering medication schedules, raising adherence risks (Sumbul-Sekerci et al., 2022; Jia et al., 2018). Functional connectivity of basal ganglia-thalamocortical structures, including the dorsomedial thalamus, caudate, and putamen, declines as cognitive status worsens (Owens-Walton et al., 2021). Emotional changes, including depression, anxiety, and apathy, also significantly impact memory (Boller et al., 1998; Broen et al., 2016; Yang et al., 2018; Weintraub et al., 2022; Hammarlund et al., 2024). Anxiety in PD engages fear-related and limbic cortico-striato-thalamo-cortical circuits, with circuitry differing from healthy populations (Carey et al., 2020). These cross-network reorganizations may reshape memory in a state-dependent manner, requiring further study (Wang et al., 2025; Longo et al., 2024).

### 5.2 State-dependent memory regulation in mental disorders

Individuals with anxiety disorders exhibit attentional biases during encoding, showing heightened vigilance to emotional cues and prioritizing negative or threatening information (Wabnitz et al., 2015; Mogg and Bradley, 2016). Encoding under threat engages the ACC more strongly, which reduces later recognition (Garibbo et al., 2019). Early biases toward negative material have been observed during encoding (Shi et al., 2024a). Anxiety also increases rumination and the retrieval of threat-related memories, sometimes promoting re-experiencing similar to PTSD (Coles and Heimberg, 2002; Lee and Fernandes, 2017; Bolton and Robinson, 2017). Neuroimaging shows that state anxiety modulates amygdala-prefrontal cortex coupling, favoring retrieval of memories congruent with the current emotional state (Garcia et al., 1999; Ganella et al., 2017). In trait anxiety, cross-network coupling varies with internal state, providing a network-level basis for SDM variability (see Subsection 3.2).

Patients with depression tend to recall more negative events, reinforcing their depressive state and creating a vicious cycle (Ellwart et al., 2003; Everaert et al., 2022). Chronic negative stress in depression is linked to disrupted hippocampal structure and functional connectivity (Zhang et al., 2024), hindering the integration of new information into existing knowledge (Sekeres et al., 2024). Memory consolidation in this context relies on reactivation of original traces (Heinbockel et al., 2024), reducing stability and weakening coherence and accuracy. Even when memories are formed, patients may not retain them long. Functional and structural changes in the hippocampus and PFC are common (Gallassi et al., 2006; Sałaciak et al., 2023). Evidence also shows network reorganization in depression, affecting systems such as the central executive network (CEN; Ho et al., 2017), anterior DMN-right FPN interaction, DMN-SN interaction (Cao et al., 2020), and emotion-related networks (Li et al., 2021). These connectivity alterations are state-dependent (Leistedt et al., 2009; Rosenbaum et al., 2015). Considering frequent selective serotonin reuptake inhibitor (SSRI) non-response (Li, 2023), understanding SDM may guide more effective treatment. NE and DA systems contribute to MDD pathophysiology (El Mansari et al., 2010), suggesting that multitarget or non-serotonergic agents may improve outcomes (Stahl et al., 2004; Daniels et al., 2024; Álvarez-Silva et al., 2024; Iqbal et al., 2024). Cognitive behavioral therapy can also improve attention and memory across emotional states by addressing negative thinking patterns (Moorey and Hollon, 2021; Bernhardt et al., 2021).

Memory impairments in PTSD include uncontrollable recollections of traumatic events, such as flashbacks, or the inability to recall certain experiences, known as dissociative amnesia (Boysan, 2016). After trauma, neuronal activity sequences often become fragmented and fail to integrate effectively into storage circuits (Kolk and Fisler, 1995; Nadel and Jacobs, 1998), leading to insufficient encoding and making the retrieval of memories difficult (Bedard-Gilligan and Zoellner, 2012). Hippocampal dysfunction contributes to these difficulties, affecting the organization and recall of traumatic event details (Acheson et al., 2012). Trauma also alters the function and structure of the amygdala in PTSD (Zhang et al., 2019; Ousdal et al., 2020). Encoding of negative information preferentially recruits the BLA (Patel et al., 2016). At the same time, reduced activity in both the hippocampus and amygdala may exacerbate trauma-related memory distortions under high stress and arousal (Hayes et al., 2011). Studies also indicate that GABAergic and glutamatergic neurons in the amygdala display distinct activation patterns (Fang et al., 2018; Zhang et al., 2019). The interplay between these regions, circuits, and neurotransmitter systems is critical for understanding PTSD-related memory impairments. Network-based analyses reveal decreased connectivity between the amygdala and PFC, supporting disrupted fronto-limbic circuits, as well as reduced connectivity within hippocampal-PFC networks (Admon et al., 2013; Spielberg et al., 2015). These network deficits can impair extinction-memory retrieval and maintenance, exacerbating intrusive recollections. PTSD is also associated with a decline in global network efficiency, particularly involving the DMN and imbalances in connectivity between the DMN and SN. These network-level disruptions contribute to fragmented memories, difficulties in memory maintenance and retrieval, and increased likelihood of intrusive re-experiencing. Considering the DMN's role in self-referential processing and recollection, and the SN's role in dynamically switching between DMN and CEN during cognitive tasks, these alterations provide a framework for testing hypotheses about memory processes in PTSD, including extinction learning, intrusive recollection, and dissociation.

### 5.3 SDM in schizophrenia

Memory impairments in schizophrenia primarily appear as deficits in memory encoding and retrieval, which may be linked to impaired novelty detection in mesolimbic circuits and reduced prefrontal and temporal lobe function (Schiltz et al., 2010). During episodic memory tasks (Ragland et al., 2009), individuals with schizophrenia show reduced PFC activation, which compromises executive function, memory encoding and retrieval efficiency, and overall information processing speed (Nathaniel-James et al., 1996). These impairments are influenced by emotional instability and high cognitive load. Emotional regulation significantly affects memory in schizophrenia, as emotional instability interferes with both encoding and retrieval through complex interactions between emotion-processing and memory systems (Herbener et al., 2007; Dieleman and Röder, 2012). Patients often show heightened sensitivity to negative emotional stimuli, which can reduce memory accuracy and introduce bias, causing inconsistencies in recalling emotional information (Sergerie et al., 2009; Walsh-Messinger et al., 2014). High cognitive load further depletes memory resources, lowering the efficiency of both encoding and retrieval (Granholm et al., 1997). During complex tasks requiring sustained attention, patients frequently struggle to allocate working memory effectively, resulting in impaired performance (Becerril and Barch, 2010; Grimes et al., 2017). Nevertheless, variations in emotional regulation and cognitive load management can partially improve memory performance. Interventions such as cognitive behavioral therapy and targeted memory training have been shown to enhance memory organization and retrieval, thereby improving memory function in schizophrenia (Bonner-Jackson and Barch, 2011; Jantzi et al., 2019).

## 6 Circuit-based neuromodulation and intervention strategies for SDM

Building on the neural circuit mechanisms of SDM described in the preceding sections, this part focuses on circuit-based neuromodulation and related intervention strategies. Electrical and non-invasive stimulation techniques such as TMS and DBS provide important tools for modulating memory-related networks, and newer approaches including transcranial direct current stimulation (tDCS) and transcranial alternating current stimulation (tACS) are expanding the range of possible applications. At the same time, closed-loop stimulation, neurofeedback, and brain-computer interface (BCI) paradigms illustrate how interventions can be tailored to ongoing brain states. Integrating neuromodulation with pharmacological and psychological treatments highlights future opportunities for multimodal and individualized therapies targeting SDM-related memory dysfunction.

### 6.1 Applications of neurostimulation technologies in memory regulation

Electrical neurostimulation technology, particularly personalized neurostimulation, is becoming important in treating neuropsychiatric disorders. However, evidence of its effectiveness in memory and state regulation varies significantly, and its safety and efficacy in human trials have not been fully validated. Further investigations are needed to deepen our understanding of its mechanisms of action, optimize stimulation protocols, and assess long-term effects of these treatments to enhance their precision and effectiveness.

To modulate brain activity and alleviate memory disorders, two widely used electrical neurostimulation techniques are TMS and DBS. TMS is a non-invasive electrical stimulation method (Roth et al., 2007; Marder et al., 2022) that can directly regulate the excitatory activity and plasticity of specific brain regions (Thomson et al., 2020; Marder et al., 2022). Multiple studies have shown that repetitive TMS (rTMS) influences memory across various states by modulating neural activity in specific brain areas (Coughlan et al., 2022), neurotransmitter levels (Claverie et al., 2024), and the functional connectivity of neural networks (Mottaghy et al., 2000). Unlike non-invasive techniques, DBS delivers continuous electrical stimulation to deep brain structures by means of implanted electrodes targeting specific regions (R. Rezai and Sharma, 2014). This technique has been used to treat various diseases, including PD, epilepsy, and mental disorders (Degirmenci, 2024). Although the mechanisms and targets of stimulation methods are different, they ultimately converge on a common brain network (Obeso et al., 2011; Siddiqi and Fox, 2022). In recent years, researchers have found that DBS can significantly improve memory-related functions by stimulating specific areas within the memory formation network, such as the hippocampus and entorhinal cortex (Khan et al., 2019).

Notably, both rTMS and DBS are influenced by the functional state of the targeted brain region, which shapes their effects on memory. rTMS may enhance or suppress memory depending on whether the cortex is in a high or low activation state, and entorhinal DBS has been observed to improve recall only when applied during neural activity patterns conducive to effective memory encoding (Silvanto and Pascual-Leone, 2008; Ezzyat et al., 2018). These findings suggest that aligning stimulation with the brain's ongoing functional state is critical for achieving consistent and effective neuromodulation outcomes. Nevertheless, rTMS and DBS do not encompass the full spectrum of circuit-based neuromodulation techniques currently under development (Chan et al., 2009; Fang et al., 2024; Wansbrough et al., 2024; Najera et al., 2025). the safety and efficacy of emerging neurostimulation paradigms still require further research (Rossi et al., 2009; Taylor et al., 2018). To better understand their potential in memory regulation and align with SDM mechanisms, it is also important to examine additional techniques that target memory-related neural networks.

### 6.2 Other circuit-based neuromodulation approaches

Beyond rTMS and DBS, several additional neuromodulation techniques have shown potential in modulating memory-related circuits and networks (Keeser et al., 2011; Antonenko et al., 2019; Mezger et al., 2020; Abellaneda-Pérez et al., 2020). tDCS (Woods et al., 2016) delivers weak electrical currents to the cortex and can alter cortical excitability (Polizzotto et al., 2020). This method has been associated with improvements in working memory, attention and executive function in conditions such as AD (Gangemi et al., 2020; Tariq et al., 2023), PD (Boggio et al., 2006) and MDD (Wolkenstein and Plewnia, 2013; Oliveira et al., 2013). Yet, clinical outcomes of tDCS remain inconsistent across studies (Suemoto et al., 2014; Bystad et al., 2016). Recent studies have employed high-definition tDCS in order to achieve more precise targeting and to monitor activity in specific circuits and networks (Rasmussen et al., 2021; He et al., 2025).

tACS applies oscillatory currents at defined frequencies and can entrain endogenous neural rhythms such as theta-gamma coupling (Diedrich et al., 2024). This mechanism has been linked to improvements in working memory (Mirjalili et al., 2025). By applying frequency-specific stimulation, tACS can modulate neural oscillations that are critical for memory formation (Shtoots et al., 2024; Paßmann et al., 2024; Zhang et al., 2025). Recent findings suggest that the after-effects of tACS depend on the ongoing state of brain activity, indicating that state-dependent plasticity may be one of the neural mechanisms influencing its efficacy (Agboada et al., 2025).

Although challenges remain in translating these novel techniques into widespread human applications (Martinez-Nunez et al., 2024), they broaden the range of circuit-based neuromodulation and provide additional avenues for interventions tailored to internal state in memory disorders.

### 6.3 Personalized neuralstimulation strategies based on neural networks

Some specially designed neurostimulation studies (Hubbard et al., 2021; Soleimani et al., 2023) have proposed that insights into the relationship between memory disorders and network imbalances may enable the development of tailored stimulation patterns that align with individual neural network patterns and physiological-psychological states. While TMS is typically designed in an open-loop mode (Marder et al., 2022), closed-loop stimulation paradigms are increasingly being investigated. These approaches integrate electroencephalography (EEG) or other neural readouts to allow for real-time adjustment of parameters based on changes in brain activity, and can therefore more precisely optimize the memory encoding process in specific brain regions (Wischnewski et al., 2024).

Recent work combining closed-loop stimulation with neurofeedback or BCI technology illustrates the potential of state-informed modulation strategies. For example, stimulation of the lateral temporal cortex guided by neural activity patterns has been shown to enhance subsequent recall ability (Ezzyat et al., 2018; Kahana et al., 2023). Adaptive methods that incorporate psychological states into stimulation protocols may improve treatment outcomes and reduce side effects (Fedotchev, 2023). At the same time, neural network models and machine learning algorithms are increasingly used to simulate and predict the dynamic behavior of the brain (Mill et al., 2022; Kurtin et al., 2023), enabling more individualized and precise regulation of memory-related processes. Together, these developments suggest that circuit-based neuromodulation, including methods described in Section 6.2, can evolve toward genuinely personalized and state-dependent interventions.

### 6.4 Future perspectives: toward multimodal and integrative therapies

While circuit-based neuromodulation has shown considerable promise in treating memory disorders, its effectiveness as a standalone intervention appears limited (Wu et al., 2025). Pharmacological treatments therefore remain indispensable, as they target neurotransmitter systems such as dopamine, norepinephrine, and GABA, which critically shape SDM processes (Bairy and Kumar, 2019; Akyuz et al., 2024). For instance, dopaminergic agents have been shown to improve executive and working memory functions through corticostriatal circuits in PD (Simioni et al., 2017). Fluctuations in dopamine activity influence whether memories are successfully encoded and retrieved under specific brain states, suggesting that pharmacological modulation can help align neural conditions with optimal memory performance (MacDonald et al., 2013).

Psychological interventions are also highly relevant, as they can reshape maladaptive emotional states and cognitive patterns, directly influencing memory encoding and retrieval biases (Hayes et al., 2023). Techniques such as cognitive behavioral therapy (Curtiss et al., 2021), mindfulness training (Hofmann and Gómez, 2017), and exposure-based therapies (Hedman et al., 2017) have been shown to enhance memory performance and emotional regulation in disorders ranging from depression and anxiety to schizophrenia. When combined with neurostimulation, these approaches may act synergistically, aligning neural modulation with cognitive and emotional states to maximize therapeutic effects (Dedoncker et al., 2021; Zhantleuova et al., 2025; Saccenti and Koster, 2024).

Integrating these pharmacological, psychological, and lifestyle approaches with neurostimulation offers a multimodal framework that targets multiple levels of neural function and behavior, creating opportunities for more precise, individualized interventions in memory disorders.

## 7 Conclusions

State dependence reveals the complexity of memory across physiological and pathological conditions and is evident at both neural and behavioral levels. In neuropsychiatric disorders, internal and external conditions such as emotional state, substance use, sleep quality, stress, and fatigue can shape each phase of memory and may exacerbate cognitive deficits.

Although SDM appears robust across many situations, several boundary conditions limit how broadly it applies. When strong external retrieval support is provided, such as in recognition tests or heavily cued recall, the reliance on internal states is reduced, and SDM effects are smaller compared with free recall (Uner and Roediger, 2022). The strength of the memory trace also plays a significant role, as SDM effects tend to weaken when memories are either highly overlearned, reaching a performance ceiling, or very weak, approaching a floor level (Petzka et al., 2021; Guskjolen and Cembrowski, 2023; Tomé et al., 2024). Additionally, time and systems-level reorganization can diminish SDM effects. Over longer delays, especially across weeks, pharmacologically induced SDM often weakens (Bardgett et al., 1996), in line with the process of systems consolidation (Dudai, 2004), which reduces the dependency of memory retrieval on encoding-state features. The specificity of the internal state is another crucial factor. When the affective/arousal state at encoding is not well-differentiated, or when mood is reinstated despite environmental changes, the apparent place-dependent effects may disappear (Corson and Verrier, 2007; Robinson and Rollings, 2010; Van Damme, 2013). Finally, task and memory-system differences indicate that SDM is not expressed uniformly across all paradigms, with some showing little or no state dependence (Radulovic et al., 2017).

Across the evidence reviewed, interactions among hippocampal, prefrontal, and amygdalar circuits, together with the organization of large-scale networks and neuromodulatory influences from DA, NE, and GABA, help explain why memory performance varies with internal state and point to interventions that are tailored to the patient's state and to the memory phase being targeted. These insights widen the range of neuromodulation approaches that act on circuits and networks in memory disorders. Although challenges remain, including individual variability and the limited translation of newer techniques into routine care, SDM provides a practical framework for designing and evaluating modulation that takes both state and phase into account. As neuroscience and artificial intelligence advance, strategies that align stimulation with a person's current state and the relevant memory phase may enable more personalized and effective treatment.
