# Supplementary material for: State-dependent memory mechanisms insights from neural circuits and clinical implications
Source: Front Cell Neurosci. 2025 Oct 30;19:1629796. doi: 10.3389/fncel.2025.1629796 (PMC12611907; doi:10.3389/fncel.2025.1629796)
Supplement: Supplementary file 1 [file Table_1.docx]

**Supplementary Table 1. Neural Circuits and Regions Underpinning State-Dependent Modulation Across Memory Phases**

| **Memory Phase** | **Neural Circuit / Region** | **Functional Role** | **Representative references** |
| --- | --- | --- | --- |
| ENCODING | Hippocampus–PFC (bidirectional connectivity) | Coordinates top-down control to tune hippocampal encoding and schema linkage | Das and Menon (2024); Su et al. (2024); Preston and Eichenbaum (2013); Sekeres et al. (2024) |
|  | Amygdala (BLA) | Signals salience and biases encoding toward emotional / threat cues | LaBar and Cabeza (2006); Patel et al. (2016); LeDoux (2003) |
|  | Hippocampus–Amygdala circuit | Binds affect with context to strengthen emotional memory traces | Phelps (2004); Madan et al. (2017) |
| STORAGE / CONSOLIDATION | PFC–Hippocampus schema network | Integrates new information into schemas, stabilizing long-term retention | Maviel (2004); Frankland and Bontempi (2005); Simons and Spiers (2003); Khan et al. (2024) |
|  | Amygdala (CeA) | Facilitates early consolidation of fear associations | Pitts and Takahashi (2011); Kim et al. (2017) |
|  | Hippocampus | Stress reduces LTP, limiting transfer to durable long-term storage | Schwabe et al. (2009); Zerbes and Schwabe (2019); Lynch (2004) |
|  | Network changes in fear memory | Humans: amygdala–dACC ↑ ; hippocampus–insula ↑ ; amygdala–mPFC ↓  Mice: thalamus–hippocampus–cortex small-world network | Feng et al. (2013); Wheeler et al. (2013) |
| RETRIEVAL | Amygdala–Hippocampus–mPFC interactions | Coordinates recall of emotional memories | Daselaar et al. (2008); Murty et al. (2010); Buchanan (2007); Oztekin et al. (2009) |
|  | Medial temporal lobe (MTL) with amygdala (arousal-related) | Arousal amplifies retrieval-related MTL activity | Dolcos et al. (2005); Madan et al. (2017) |
|  | Fear retrieval microcircuits (BLA→PL; vHPC→PL) | Opposing inputs to PL gate fear expression at recall | Sotres-Bayon et al. (2012); Sierra-Mercado et al. (2011) |
|  | ACC | Supports retrieval of remote contextual fear memories | Frankland et al. (2004) |
|  | Fear extinction pathways (IL→BLA; IL→Re→BLA); Re links mPFC–HPC | Inhibits fear expression and relays mPFC–hippocampus communication | Li et al. (2025); Vafaei et al. (2022); Silva et al. (2021); Viena et al. (2018); Shoob et al. (2023) |

Legend: Major brain regions and pathways implicated in state-dependent effects at each phase. Citations match Section 2 in the main text. This table complements Table 1 (behavioral effects). Abbreviations: BLA, basolateral amygdala; CeA, central amygdala; PL, prelimbic cortex; IL, infralimbic cortex; Re, nucleus reuniens; MTL, medial temporal lobe; ACC, anterior cingulate cortex; dACC, dorsal anterior cingulate cortex; PFC, prefrontal cortex; mPFC, medial prefrontal cortex; vHPC, ventral hippocampus. Symbols: “→” denotes directional pathway; “↑/↓” denote increased/decreased functional connectivity (FC) between the indicated regions.
